# Habitat reconstruction for the Late Pleistocene Siberian saber-toothed cat *Homotherium* using microphytofossils

**DOI:** 10.1038/s41598-025-26872-7

**Published:** 2025-11-28

**Authors:** A. V. Lopatin, D. A. Lopatina, O. G. Zanina, A. V. Protopopov, A. I. Klimovsky

**Affiliations:** 1 Borissiak Paleontological Institute, Russian Academy of Sciences, Moscow, Russia; 2 Geological Institute, Russian Academy of Sciences, Moscow, Russia; 3 Institute of Physicochemical and Biological Problems in Soil Science, Russian Academy of Sciences, Pushchino, Moscow Region Russia; 4 Academy of Sciences of the Republic of Sakha (Yakutia), Yakutsk, Russia

**Keywords:** Ecology, Evolution, Ecology, Environmental sciences

## Abstract

The frozen mummy of a saber-toothed cat *Homotherium latidens* cub was found in the Upper Pleistocene permafrost of the Yedoma deposits on the Badyarikha River (the right tributary of the Indigirka River) in the northeast of Yakutia (East Siberia, Russia). We present the results of a study of organic microfossils (pollen, spores, plant detritus, etc.) and phytoliths from a deposit sample collected at the site of the discovery of the frozen mummy. Habitat of the Siberian *Homotherium* in the Badyarikha River region is reconstructed as a floodplain mature larch forest and mesic sedge-grass-forb meadows.

## Introduction

**Fig. 1:**
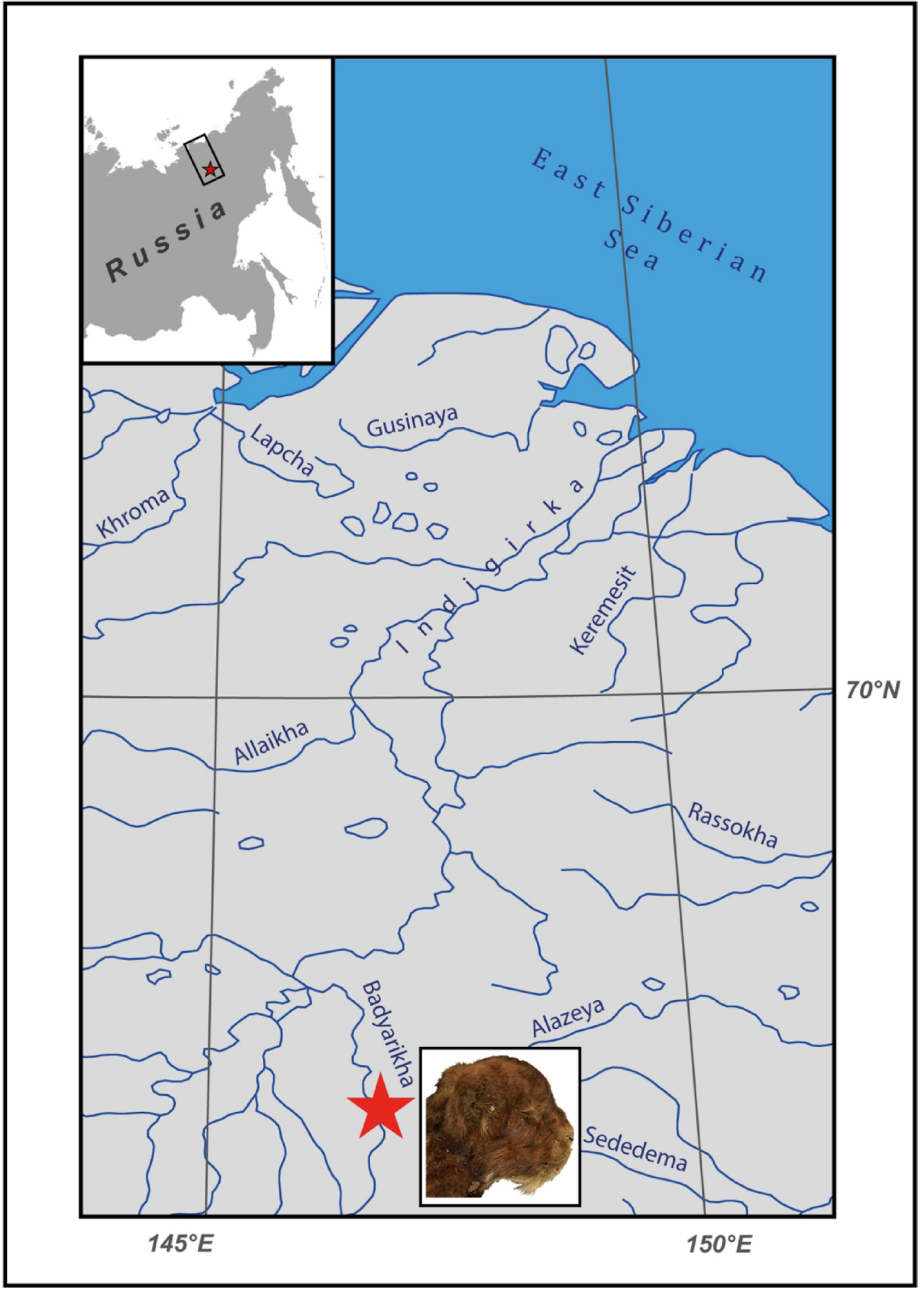
Geographical location of the Badyarikhskoe locality (star) yielded the Late Pleistocene frozen mummy of the saber-toothed cat cub (*Homotherium latidens*, photo of the head, right lateral view); Russia, Republic of Sakha (Yakutia), Indigirka River basin, Badyarikha River.

A frozen mummy of a three-week-old cub of a saber-toothed cat *Homotherium latidens* (Owen, 1846) was found in the Abyisky ulus of the Republic of Sakha (Yakutia) in 2020 and described in 2024 ^1^. The Badyarikhskoe locality takes place on the Badyarikha River (right tributary of the Indigirka River, Yana-Indigirka Lowland; 67°41ʹ14ʹʹ N, 146°46ʹ13ʹʹ E) (Fig. 1). Frozen deposits containing large ice lenses are exposed in the cliff of the river terrace. Numerous bones of the Late Pleistocene mammals are collected from the loess-like loams of the Yedoma horizon in this locality, including *Canis lupus* Linnaeus, 1758, *Vulpes vulpes* (Linnaeus, 1758), *Ursus arctos* Linnaeus, 1758, *Gulo gulo* (Linnaeus, 1758), *Panthera spelaea* (Goldfuss, 1810), *Mammuthus primigenius* (Blumenbach, 1799), *Equus lenensis* Russanov, 1968, *Coelodonta antiquitatis* (Blumenbach, 1799), *Rangifer tarandus* (Linnaeus, 1758), and *Bison priscus* (Bojanus, 1827) ^2^. Radiocarbon dating of the mummy (based on wool) is 31,808 ± 367 years BP, calibrated as 35,471–37,019 years cal BP ^1^.

The Badyarikha mummy contains the head and the anterior part of the body^1–3^. In addition to the collection number (DMF AS RS, no. Met-20-1), here we also provide the informal nickname of this specimen, “Meten,” from the name of the oxbow lake near the site of the discovery of the mummy. The external appearance of the scimitar-toothed cat cub has been studied by direct observation. The shortened body with very massive neck and elongated forelimbs of the studied cub reflect the characteristic features of the adult *Homotherium* individuals, known from skeletal morphology. The enlarged mouth and high upper lip of the juvenile are related with the development of hypertrophied upper canines and very large incisors in adults. A number of observed external characteristics (wide paws, small low auricles) may be associated with cold snowy habitats. However, no special studies of the environment of the Siberian *Homotherium* have been conducted to date.

This paper presents the results of a study of organic microfossils (pollen, spores, plant detritus, fungal spores, algae remains) and siliceous microfossils (phytoliths and sponge spicules) from a deposit sample collected at the site of the discovery of the frozen mummy. The aim of this work is to provide the detailed reconstruction of the vegetation and landscape of the Siberian scimitar-toothed cat habitat.

## Material and methods

**Fig. 2:**
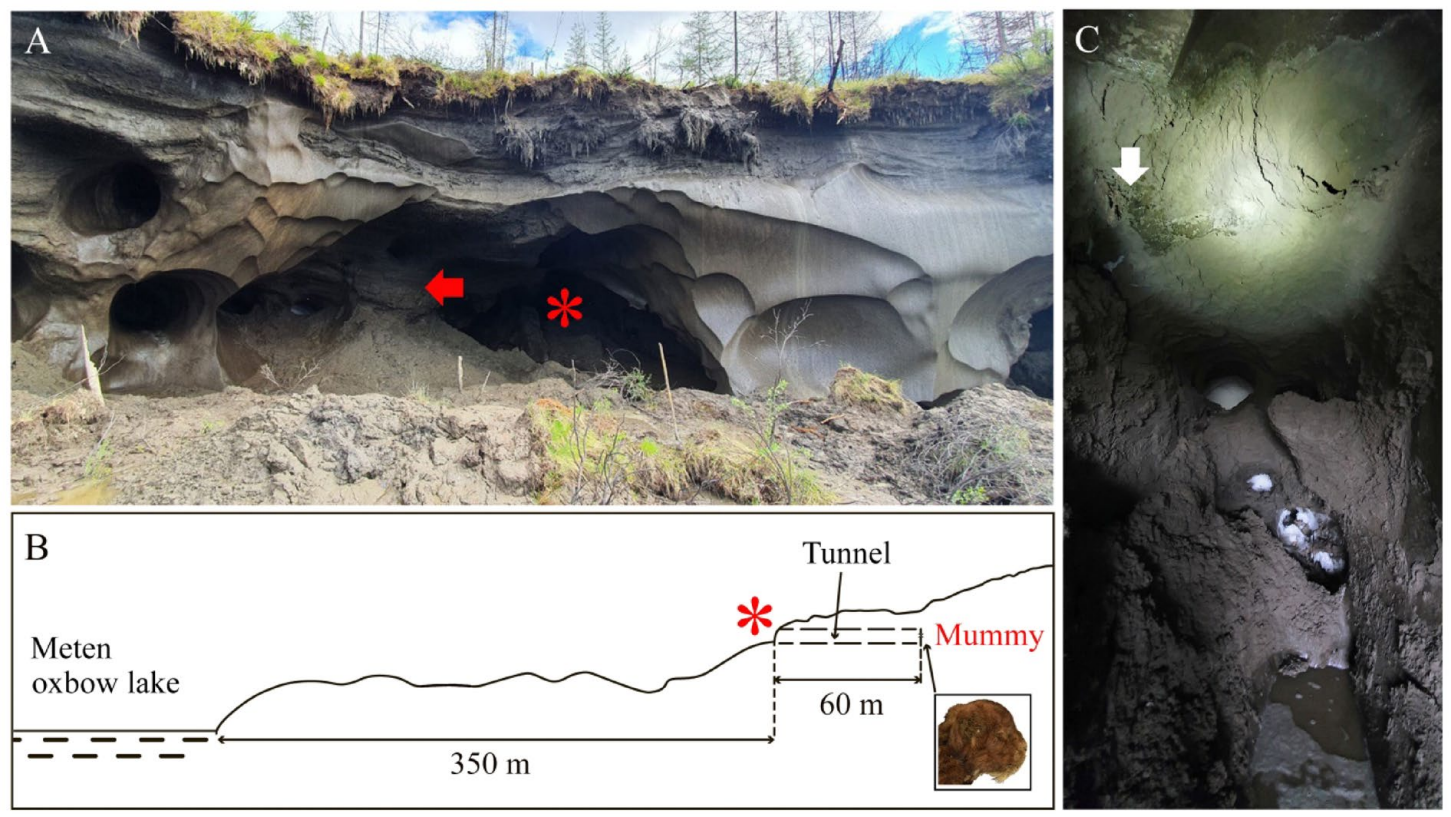
Upper Pleistocene Badyarikhskoe locality: (**A**) view of outcrop; (**B**) schematic profile with the designation of the washed tunnel and the location of the find of the frozen mummy of the saber-toothed cat cub (*Homotherium latidens*); (**C**) view of the site of the frozen mummy finding. Designations: red asterisk, entrance to washed tunnel; red arrow, exposed investigated fossil-bearing layer; white arrow, sampling place.

The Badyarikhskoe locality (Badyarikha site 1 “Meten”) is situated in the upper reaches of the Badyarikha River, near the Meten oxbow lake ^4^. The place where the mummy was found is located inside a tunnel washed out in the bluff by water pumps during the extraction of mammoth tusks (Fig. 2).

The locality is situated 350 m from the bank of the oxbow lake. This is a retrogressive thaw slump about 80 m long; on the left there is a large ice wedge, breaking the frozen deposits into blocks, in one of which a tunnel up 60 m deep and about 2 m high has been washed out (Fig. 2, A, B). The height of the outcrop is about 20 m (58 m above the lake water level), and the modern active layer reaches a maximum thickness of about 0.5 m. The permafrost sediments are mainly finely laminated alluvial loams; they have been characterized recently ^4^. The fossil bones are located on the loam itself, they fall out as a result of seasonal thawing of the cryogenic beds. The mummy was found in the deepest part of the washed tunnel, at a height of about 1.4 m from its bottom (Fig. 2B). The sampling place is located directly under the mummy (Fig. 2C); the investigated fossil-bearing layer is exposed in the lower part of the outcrop (Fig. 2A). The mammoth tusk samples apparently related to these deposits have been dated as 33,748–35,924 and 37,097–38,336 years cal BP ^4^.

**Fig. 3:**
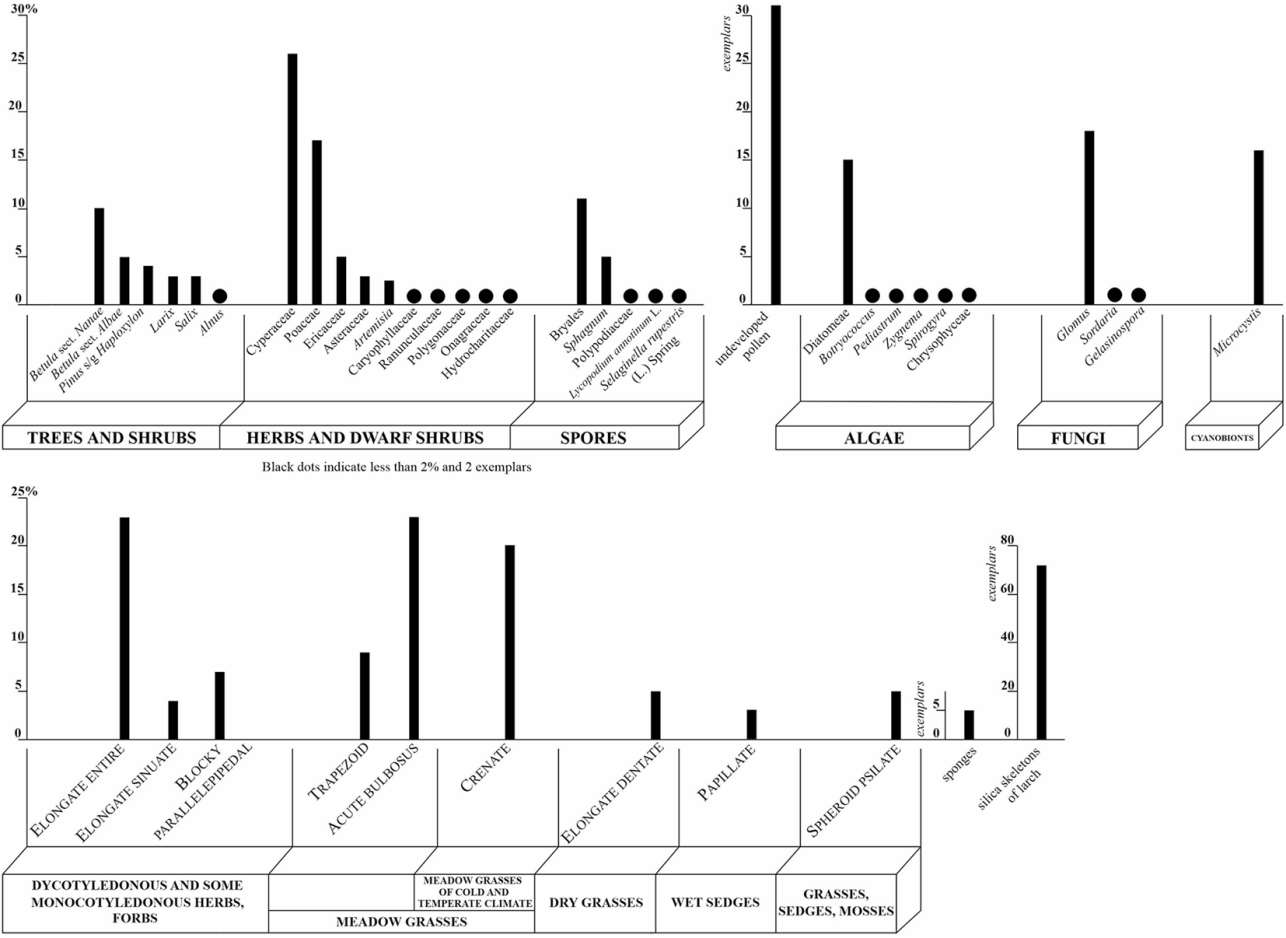
Content of microfossils from the deposit sample collected at the site of the discovery of the frozen mummy of the saber-toothed cat *Homotherium latidens* cub; Russia, Republic of Sakha (Yakutia), Indigirka River basin, Badyarikhskoe locality; Upper Pleistocene.

The sample for palynological and phytolith analyses was processed using the separation method proposed by V.P. Grichuk ^5^ without treating the macerate with acetolysis mixture. Water-free glycerol was used for preparation of the microscopic slides. Palynomorphs were identified using LOMO MIKMED-2 microscope at magnification of ×400. The microscopic analyses revealed sufficiently high pollen concentration and good preservation of palynomorphs to allow the counting 500 grains. The results of the palynological analyses are presented as percentage diagrams, where each pollen and spore taxon is given as a percentage of the sum of all identified palynomorphs, which was taken as 100% (Fig. 3). The quantity of the redeposited palynomorphs (*Ulmus* and *Juglans* occurred as single grains, presumably redeposited from the Paleogene/Neogene), undeveloped pollen and so-called non-pollen palynomorphs (fungal spores, remains of algae and cyanobionts) was not included in the calculated sum. Their content per 500 specimens of spores and pollen was counted.

Phytolith analysis was conducted along with palynological in the same material. The content of phytoliths was estimated separately (based on the calculated sum equal to 300). The names of phytolith forms were accepted according to ICPN 2.0 ^6^. The relationship between phytolith morphotypes and their biocenotic characteristics was done according to N.E. Ryabogina et al. ^7^, with modifications. The presence of silica skeletons of larch and plant debris was also assessed.

Images of microphytofossils were taken using the Olympus BX53-F2 light microscope and the TESCAN Vega 3 LSU scanning electron microscope (SEM). The light microscope images were captured using the MSH-C camera with MShot Image Analysis System V1.1 and UPlanFL N 40×/0.75 Ph2 ∞/0.17 microscope objective lens. The images have the following characteristics: 2048 × 2048 matrix sizes, 120 dpi resolution, 24-bit RGB, gamma 1. SEM images were taken with the VEGA3 LMU camera and BSE detector, using 20 kV accelerating voltage, 15 mm WD, about 700 µm view field, 1028 × 1530 matrix sizes, 183 dpi resolution, and 16-bit. All the images were displayed with no level adjustment. The final figures were assembled in Adobe Photoshop program.

Abbreviations: DMF AS RS, Department for the Study of Mammoth Fauna, Academy of Sciences of the Republic of Sakha (Yakutia), Yakutsk, Russia.

## Results

**Fig. 4:**
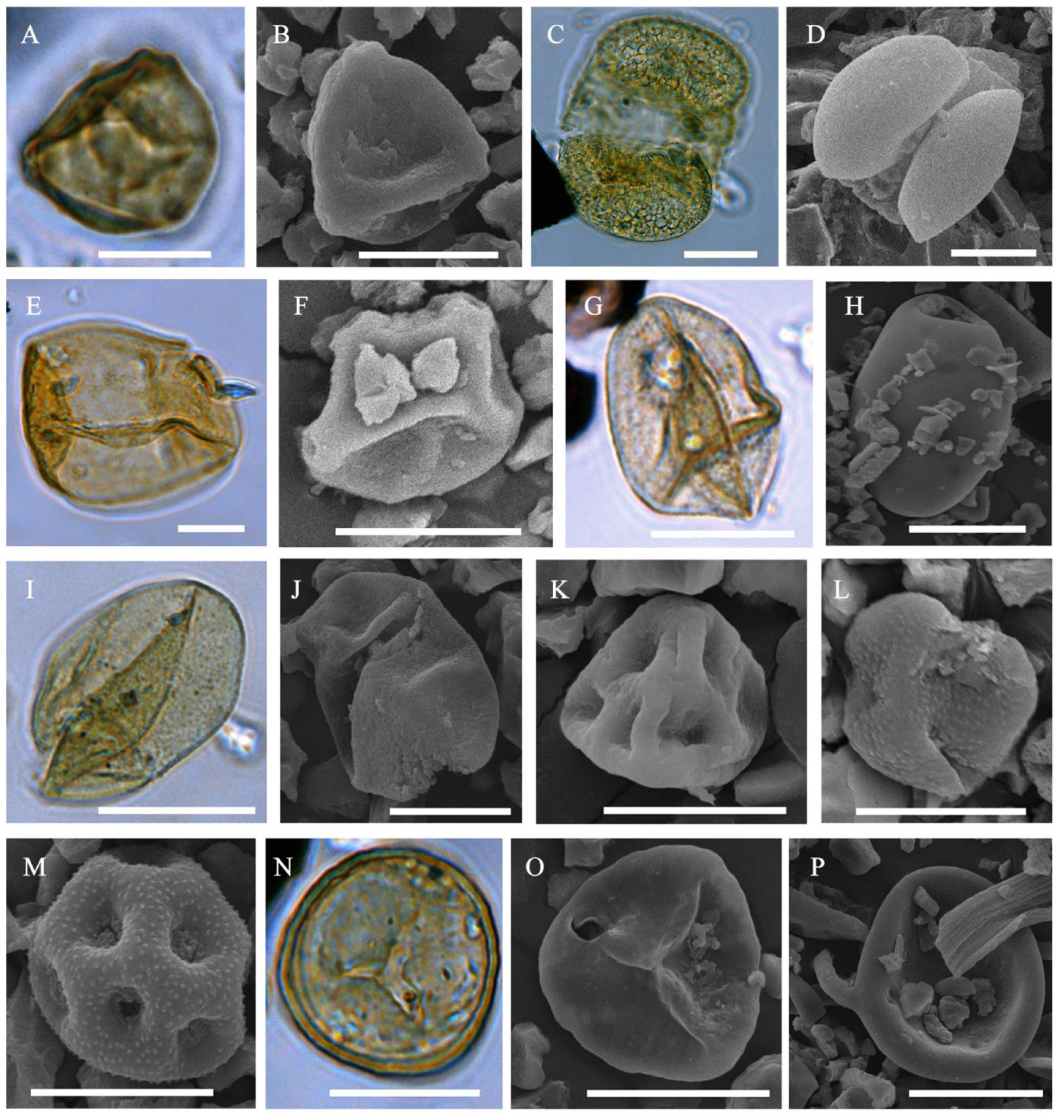
Microphytofossils from the deposit sample collected at the site of the discovery of the frozen mummy of the saber-toothed cat *Homotherium latidens* cub; Russia, Republic of Sakha (Yakutia), Indigirka River basin, Badyarikhskoe locality; Upper Pleistocene: (**A**, **B**) *Betula* sect. *Nanae*; (**C**, **D**) *Pinus* s/g *Haploxylon*; (**E**) *Larix*; (**F**) *Alnus*; (**G**, **H**) Poaceae; (**I**, **J**) Cyperaceae; (**K**) Ericaceae; (**L**) Asteraceae; (**M**) Caryophyllaceae; (**N**, **O**) *Sphagnum*; (**P**) *Glomus*. Light microscope images (in color) and SEM images. Scale bar is 20 µm.

The sample yielded a pollen record (Figs. 3, 4) dominated by herb and dwarf shrub pollen (55%) with Cyperaceae (26%) and Poaceae (17%) being most abundant. In this group, the minor components are not diverse; they include Ericaceae, *Artemisia*, Asteraceae, Ranunculaceae, Caryophyllaceae, Polygonaceae, Onagraceae, *Valeriana*, and Hydrocharitaceae. Tree and shrub pollen group (27%) is represented mainly by *Betula* sect. *Nanae* with a small proportion of *Larix*, *Pinus* s/g *Haploxylon*, *Betula* sect. *Albae*, *Alnus*, and *Salix*. Spores are not abundant (18%) and include Bryales, *Sphagnum* and minor occurrence of *Selaginella rupestris* (L.) Spring, Polypodiaceae, and *Lycopodium annotinum* L.

The undeveloped pollen (immature pollen of unclear systematic position, presumably related to forbs) was established in the amount of 31 specimens for 500 counted palynomorphs.

Algae are represented by freshwater diatoms with mostly broken valves, scraps of green algae colonies of *Botryococcus* and *Pediastrum*, cysts of Characeae algae *Zygnema* and *Spirogyra*, stomatocysts of Chrysophyceae algae. Cyanobiont cells of *Microcystis* are detected in noticeable amount. Fungi were also observed in the macerate. *Glomus* was the most common among them, coprophilous fungi *Sordaria* and *Gelasinospora* occurred as single specimens; last genus also is carbonicolous and often grow on charred wood ^8^.

**Fig. 5:**
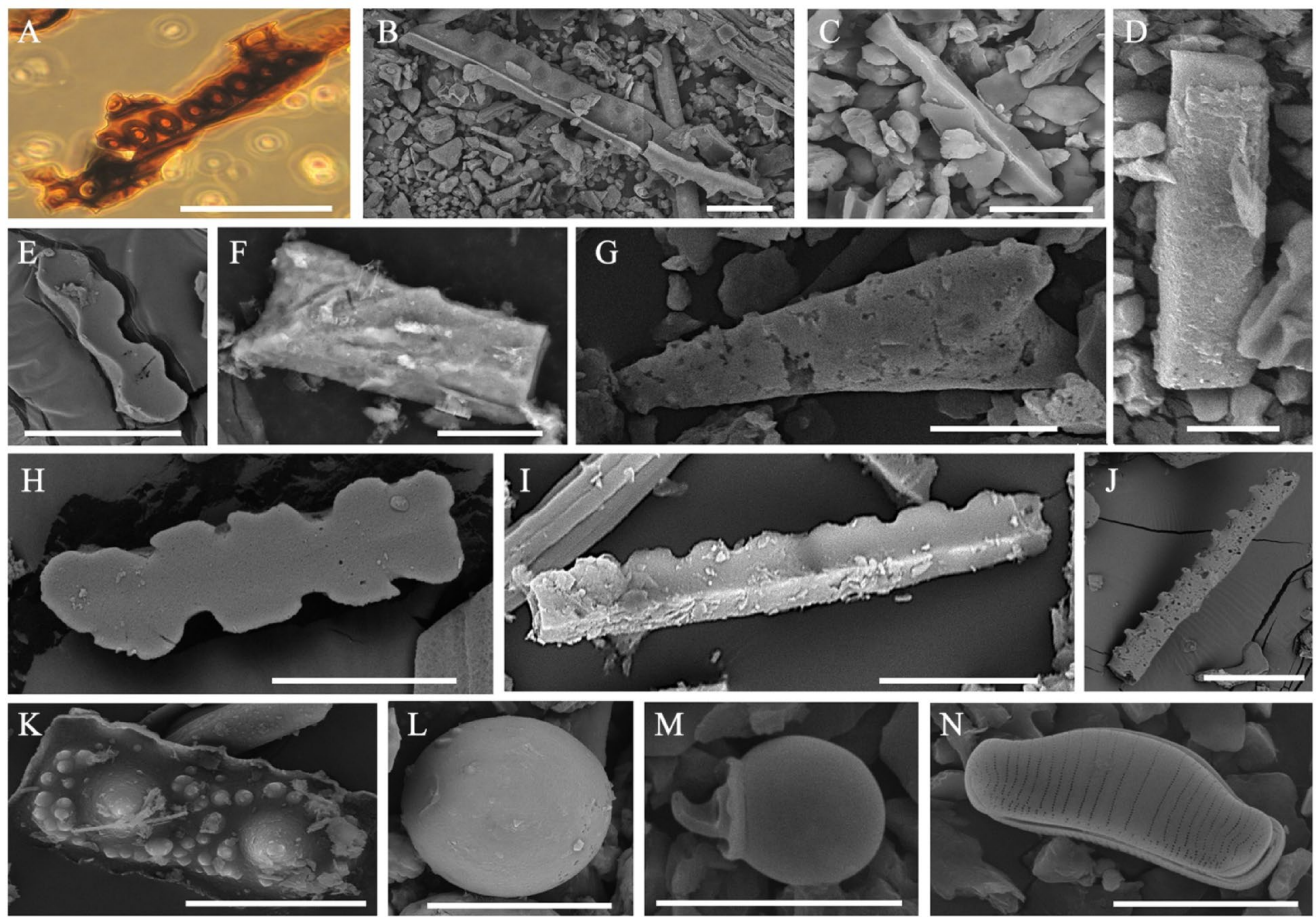
Microphytofossils from the deposit sample collected at the site of the discovery of the frozen mummy of the saber-toothed cat *Homotherium latidens* cub; Russia, Republic of Sakha (Yakutia), Indigirka River basin, Badyarikhskoe locality; Upper Pleistocene: (**A**–**C**) silica skeletons of larch; (**D**–**L**) phytoliths: (**D**) longate entire, (**E**) longate sinuate, (**F**) Bloky Parallelepipedal,(**G**) Acute bulbosus,(**H**) Trapezoid, (**I**) Crenate,(**J**) Elongate dentate,(**K**) Papillate, and(**L**) pheroid psilate; (**M**) Chrysophyceae stomatocyst; (**N**) diatom alga *Eunotia praerupta* Ehrenberg, 1843. Light microscope image (in color) and SEM images. Scale bar is 20 µm.

The main content of the phytolyth spectrum consists of Elongate entire, Crenate and Acute bulbosus forms. In quantities not exceeding 7%, Trapezoid, Elongate dentate, Elongate sinuate, Blocky parallelepipedal, Spheroid psilate, and Papillate were determined (Figs. 3, 5).

## Discussion

### Reconstructed vegetation

Analysis of spores, pollen and phytoliths allows to reconstruct predominance of herbaceous communities (sedge, sedge-grass, and grass-forb). The families of herbs and dwarf shrubs identified by pollen are represented by a significant number of species of different ecological confinement. It suggests the existence of meadows with varying moisture degrees. The mass content of pollen of grasses and sedges and the forms of phytoliths Crenate and Acute bulbosus, characteristic of meadow grasses, is evidence of their predominance in the vegetation cover.

The presence of forb pollen in the spectrum is low. This may be due to the low pollen productivity of these plants under severe climatic conditions and their transition to vegetative reproduction ^9^. The high participation of forbs in the vegetation is emphasized by the noticeable amount of undeveloped pollen and phytoliths Elongate entire that are typical for dicotyledonous herbs and partly grasses. The mentioned forms of phytoliths are formed in the columnar parenchyma of leaves of dicotyledonous grasses, although some of them could have been formed by monocotyledonous plants. It is possible that the predominance of Elongate entire forms is due to their solid structure facilitated their preservation in sediments ^10^. These forms are poorly informative, but in this case they emphasize the significant role of meadow forbs and grasses in the vegetation. The presence of the Elongate sinuate and Bloky parallelepipedal forms confirms this conclusion.

Albeit insignificant, the finds of Papillate phytoliths of wetland sedges suggest the presence of tundra meadows on moist substrates. The existence of such habitats found on the banks of lakes and rivers and in lowland meadows is also indicated by *Sphagnum* spores and minor herb taxa *Valeriana* and Ranunculaceae, growing in the conditions of sufficient or excessive moisture. These conditions could have developed as a result of an increase in the thickness of seasonally thawed layer due to the melting of polygonal ice wedges. The result of the latter is also activation of slope and erosion processes. The presence of scree slopes, well-drained and/or disturbed and undeveloped soils is diagnosed by the findings of sagebrush *Artemisia* and rock spike-moss *Selaginella rupestris*, phytoliths Elongate dentate, characteristic for grasses growing on dry substrates and also *Glomus* fungi which are indicator of active soil erosion and the prevalence of aeolian processes in open landscapes ^8^.

Due to its low productivity, poor distribution and easy destructibility ^11^ even small amount of larch pollen is conventionally taken to indicate sparse trees or small patches of larch forest in the reconstructed vegetation. Large number of specific forms typical for *Larix* wood found in plant detritus is additional evidence for larch in the region. These forests were contributed by shrub birch, which also can form streamside thickets in tundra. Mesic forbs (Ranunculaceae, Ericaceae) and mosses were in the ground cover of the larch forest.

Based on the small pollen content of *Alnus*, *Pinus* s/g *Haploxylon* (the latter indicates the presence of the shrub *Pinus pumila* (Pall.) Endl. in Quaternary records from northeastern Asia), and *Betula* sect. *Albae* from the tree species of birch it is difficult to conclude about their roles in the vegetation. Trees and shrubs of above genera produce a large amount of highly wind transportable pollen therefore it can reflect long-distance pollen transport, depending upon atmospheric conditions. The low percentages of mentioned pollen also could suggest the rare occurrence of scattered thickets of alder and occasional dwarf pine and birches in the understory of larch forests.

The amount of willow pollen in the spectrum is small. Due to entomophily and dioecy, its content even in subfossil spectra is low, provided that the sample is collected directly near the plant ^12^. Willow may have formed thickets along river banks and on lower slopes.

Trace amounts of dung-inhabiting fungal spores of *Sordaria* and *Gelasinospora* likely indicate the presence of grazing animals. *Gelasinospora* also provides the evidence of fires.

The spectrum is interpreted as a mix of a floodplain mature larch forest restricted to the valley bottom and lower elevation slopes and mesic sedge-grass-forb meadows with a mosaic of moist to xeric habitats.

The sediments were accumulated under wetter conditions as evident by notable amounts of remains of cyanobionts, diatoms, sponge spicules, finds of green algae *Botryococcus* and *Pediastrum*, charophytic algae *Zygnema* and *Spirogyra*, stomatocysts of Chrysophyceae algae, phytoliths of wetland sedges, pollen of water plants and plants of moist substrates.

These environments could have existed in shallow ponds or swamps, formed under the conditions of increased moisture from groundwater in the presence of waterproof of permafrost soils in the relief depressions or on a flooded river bank.

Our conclusions are quite consistent with the results of macrophytofossil analysis of colluvium deposits in the studied locality, which allowed to identify *Salix* sp., *Larix sibirica* Ledeb., *Betula* sp., *Cladonia portentosa* (Dufour) Coem., and *C. rangiferina* (L.) Weber ex F.H. Wigg. and the analyses of fossil humus and mammoth coprolites, in which *Bryum* sp., Polypodiaceae, cf. *Carex* sp., *Eleocharis* sp., and cf. *Cicuta virosa* L. were reported ^4^.

The palynological data from the MIS 3 deposits of the Badyarikha River sites and other localities of the central Indigirka basin ^4^ document changes in the landscape cover over the past about 50 ka. They indicate taiga forest and parkland-steppe ecosystems within the central Indigirka basin during the warmest MIS 3 interstadial interval (~ 50–45 ka BP). At a later stage (after ~ 40 ka BP), the ecosystems of the forested valleys transformed into a larch-dominated forest-tundra, with thickets of alder, willow, and dwarf birch, floodplain meadows, marshes, and herbaceous grasslands, and our results show that the Siberian *Homotherium* existed in such environments. At the end of the interstadial (~ 30–25 ka BP), the shrub- and grass-dominated herb-tundra-steppe was formed in the region ^4^.

Palynological data for the second half of MIS 3 (ca. 42,000–30,000 years cal BP) suggest that the landscape on the territory of Yana-Indigirka-Kolyma lowland supported open areas with herb-dominated associations. Plant communities were represented by forb-grass-sedge, sagebrush-grass-sedge, grass-sagebrush-forb associations, xeropetrophyte associations, tundra associations on moist soils along riverbanks and in the river valleys and willow thickets ^13^. *Larix* forests were widely present from Priokhot’ye to the northern Yana-Indigirka-Kolyma lowlands in these times ^14^. These forests were probably open woodlands restricted to lower elevations in the river valleys or isolated stands in favorable sites.

### *Homotherium***habitats**

The habitats of *Homotherium* were very diverse, and the range of this genus included Africa, Eurasia and America ^15^. Judging by the structure of the skeleton, which reflects a cursorial adaptation (at moderate speeds for large distances), *Homotherium* did not live in dense forests, but preferred more open spaces ^16^. Analyses of dental microwear textures and stable isotopes of tooth enamel indicate that this likely social predator preferred soft and tough flesh of open-habitat prey, including bison, horses, and juvenile mammoths ^17,18^.

**Fig. 6:**
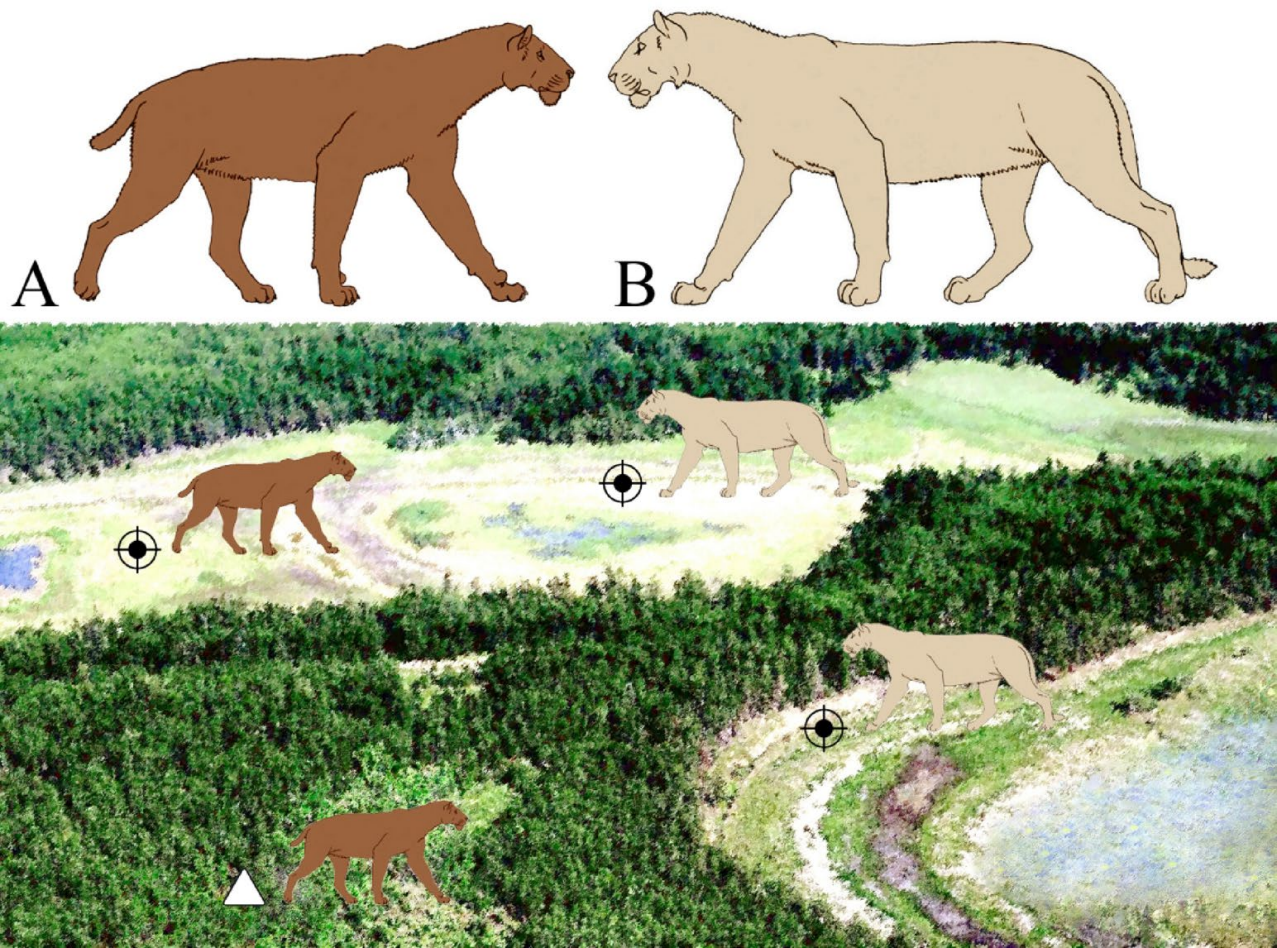
Reconstructed habitat pattern of the co-existed Late Pleistocene saber-toothed cat, *Homotherium latidens* (**A**), and cave lion, *Panthera spelaea* (**B**), on Badyarikha River in northern Yakutia. Designations: black crosshair, hunting grounds of both homotheres and cave lions; white triangle, den sites of homotheres. Not to scale. The extinct felid outline images after Antón et al.^16^.

Interesting results were obtained by analyzing the evolutionary history of late representatives of *Homotherium* in context of their possible competitive relationships with other large predators ^19,20^. In the Pleistocene of Europe, the main competitor of *Homotherium* for hunting grounds and prey was the cave lion, *Panthera spelaea*, which was somewhat larger than the modern lion. The cave lion’s size, strength, and social lifestyle gave it a dominant position in the guild of large carnivores. It is really unknown how intense was this competition (scimitar-toothed cats were probably diurnal hunters ^17^, while cave lions were more active at night). Nevertheless, these similar-sized species (with weights of 100–220 kg for the late representatives of *Homotherium*, and 140–300 kg for *Panthera spelaea spelaea*, see Fig. 6) clearly interacted aggressively, and *Homotherium* probably found a way to reduce this competitive pressure by avoiding lion favorable habitats ^20,21^. The cave lion inhabited predominantly plains and river valleys ^22^. Open habitats were preferable for *Homotherium* hunting technique, but competition from the cave lion have forced it to seek moderate cover, which was provided by a mosaic of woodlands and grasslands ^16^. In northern Siberia, these mosaic landscapes were the arctic tundra steppe and northern forest-tundra areas, where low larch formed sparse forests with patches of meadows ^13,14^.

**Fig. 7:**
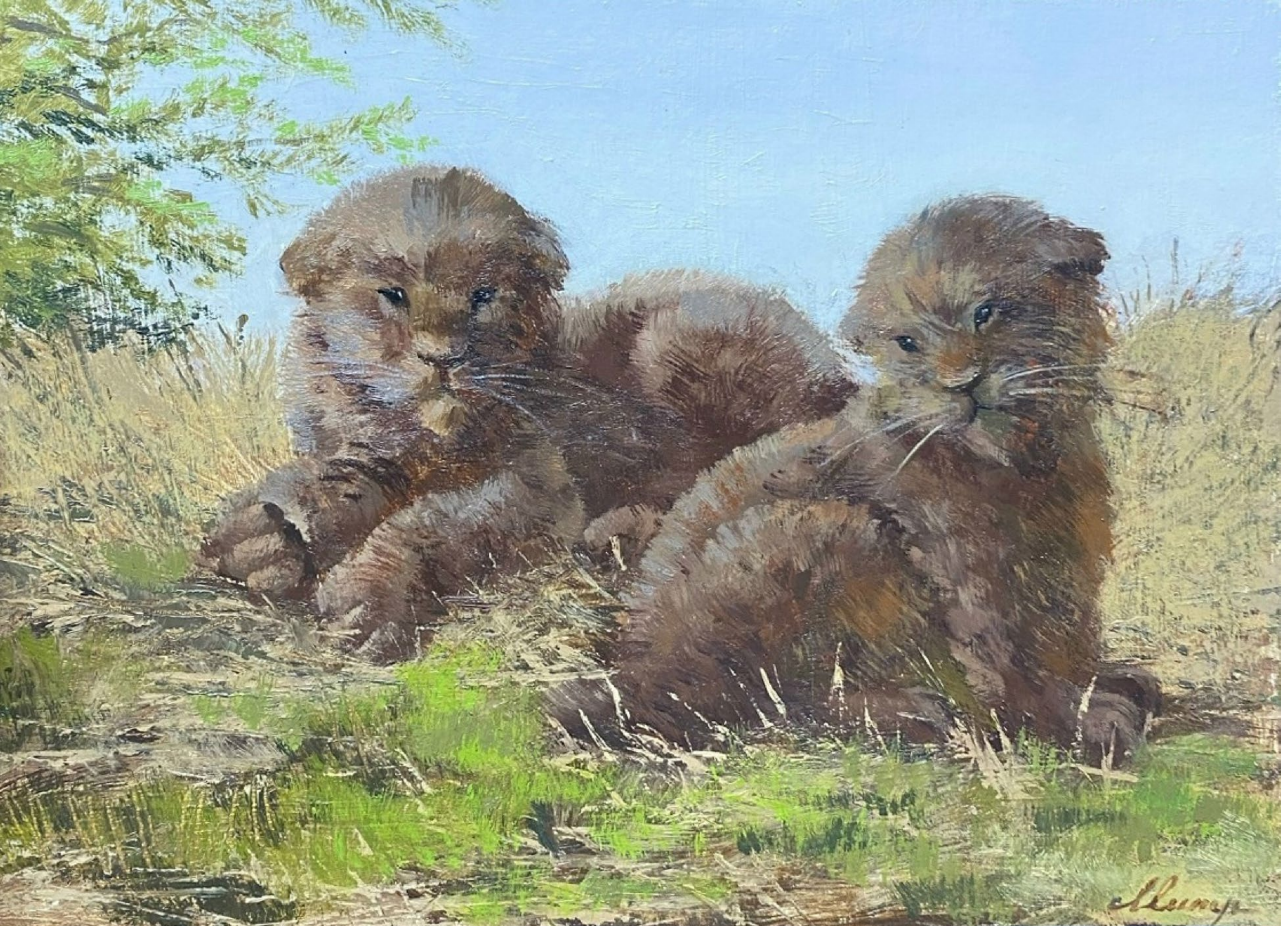
Saber-toothed cat’s cradle: Siberian *Homotherium* cubs at the edge of a larch forest. Artistic reconstruction by P. Mitroshkina (with scientific consultation by A.V. Lopatin).

In the Late Pleistocene of Eurasia, representatives of *Homotherium* may have existed at low population densities. This effectively put them below the “fossil detection threshold” and left very few remains in the fossil record ^23^. The same has been proposed to explain the low prevalence of relict *Homotherium* in the Americas ^24,25^. These ranges were limited to small habitats and may even have been refugia. Anyway, the discovery of the Badyarikha saber-toothed cat mummy proves that during the Karginsky interval, correlated with MIS 3 (at least until around 37 ka BP) the *Homotherium* population survived in this region of northern East Siberia. Judging by found bone remains, the cave lion lived there at the same time. Consequently, these two large felid predator species co-existed in this region. The find of a three-week-old cub, indicating the presence of a den, identifies this area as the domain of *Homotherium*. It can be assumed that this situation was related to local environments. Probably, such a suitable habitat was the reconstructed mature larch forest, which served as a shelter for adult saber-toothed cats and a hidden cradle for their cubs (Fig. 7).

## Conclusion

The investigation of organic microfossils (pollen, spores, plant detritus, etc.) and phytoliths from a deposit sample collected at the site of the discovery of the frozen mummy of the saber-toothed cat *Homotherium latidens* cub found in the Upper Pleistocene deposits on the Badyarikha River in northeast Yakutia revealed to reconstruct the Siberian *Homotherium* habitat as a floodplain mature larch forest and mesic sedge-grass-forb meadows. Perhaps such relatively closed local habitats allowed these saber-toothed cats to avoid intense competition and direct aggressive interactions with co-existed cave lions.

The reconstruction of floodplain larch forest and mesic sedge-grass-forb meadows is well supported by the palynological and phytolith evidence. But at the same time, our conclusions concerning the role of such habitats in reducing competition with cave lions and serving as shelters for *Homotherium* cubs are preliminary and require additional supporting data.

## Data Availability

All data generated or analysed during this study are included in this published article. Please contact Prof. A.V. Lopatin in case of any queries regarding this study.
